# Human genetics in troubled times and places

**DOI:** 10.1186/s41065-017-0042-4

**Published:** 2017-08-03

**Authors:** Peter S. Harper

**Affiliations:** University Research Professor (Emeritus) in Human Genetics, Institute of Medical Genetics, School of Medicine, Cardiff University, Heath Park, Cardiff, CF14 4XN UK

**Keywords:** History, Human, Medicine, Medical, Genetics, War, Russia, ethics

## Abstract

The development of human genetics world-wide during the twentieth century, especially across Europe, has occurred against a background of repeated catastrophes, including two world wars and the ideological problems and repression posed by Nazism and Communism. The published scientific literature gives few hints of these problems and there is a danger that they will be forgotten.

The First World War was largely indiscriminate in its carnage, but World War 2 and the preceding years of fascism were associated with widespread migration, especially of Jewish workers expelled from Germany, and of their children, a number of whom would become major contributors to the post-war generation of human and medical geneticists in Britain and America. In Germany itself, eminent geneticists were also involved in the abuses carried out in the name of ‘eugenics’ and ‘race biology’. However, geneticists in America, Britain and the rest of Europe were largely responsible for the ideological foundations of these abuses.

In the Soviet Union, geneticists and genetics itself became the object of persecution from the 1930s till as late as the mid 1960s, with an almost complete destruction of the field during this time; this extended also to Eastern Europe and China as part of the influence of Russian communism. Most recently, at the end of the twentieth century, China saw a renewal of government sponsored eugenics programmes, now mostly discarded.

During the post-world war 2 decades, human genetics research benefited greatly from recognition of the genetic dangers posed by exposure to radiation, following the atomic bomb explosions in Japan, atmospheric testing and successive accidental nuclear disasters in Russia.

Documenting and remembering these traumatic events, now largely forgotten among younger workers, is essential if we are to fully understand the history of human genetics and avoid the repetition of similar disasters in the future. The power of modern human genetic and genomic techniques now gives a greater potential for abuse as well as for beneficial use than has ever been seen in the past.

## Background

*‘We shall go to the pyre, we shall burn, but we shall not retreat from our convictions. I tell you, in all frankness, that I believed and still believe and insist on what is right, and not only believe - because taking things on faith in science is nonsense - but also say what I know on the basis of wide experience. This is a fact, and to retreat from it simply because some occupying high posts desire it, is impossible.’*

From speech of N.I. Vavilov to All Union Institute of Plant Breeding, USSR. March 1939 (from Medvedev [1]).

This quotation from Nikolai Vavilov, at the height of the greatest crisis that modern genetics, including human genetics, has ever faced during its existence, may seem now to be an echo from the distant past, but the issues that Vavilov, perhaps Russia’s greatest scientist ever, had to confront are as relevant to us today as they were in the troubled times of Soviet Russia under Stalin’s ‘great terror’. I shall return to the story of the destruction of genetics in Russia later, but it is only one, though probably the most extreme, of a number of traumatic events in the history of the field.

Over the period of little more than a century since the rediscovery of Mendel’s work in 1900, genetics, especially human genetics, has rarely had a peaceful existence. From the rapid pace of scientific and medical advances one would not think that this was the case, nor does the published literature in journals and books indicate the successive crises that often involved its workers, both professionally and in their personal lives. It is only through the less scientific writings by or about these workers, and in recent years through recorded interviews with those involved, that one begins to appreciate the traumatic background to much of their work. I attempt here to document what can only be small fragments of their stories as a belated tribute to their experiences and sufferings, and those of others across the world who did not survive to pursue their work further.

## World war 1

The first decade after the rediscovery of Mendel’s work in 1900 saw a sudden burst of new insights into human genetics, even though Mendel’s work itself, and that of his discoverers, had been primarily on plants. The enthusiasm of William Bateson in England and his links with physicians involved with hereditary disorders soon established the universality of Mendelism, and the medical world was quick to take up the field. Archibald Garrod’s studies of the inheritance of alkaptonuria [2] (in collaboration with Bateson) and of other inherited metabolic disorders established the concept of ‘inborn errors of metabolism’ (Garrod [3]), while numerous previously published family studies of inherited eye, skeletal and neurological conditions now fell into place as following specific patterns of mendelian inheritance, forming the foundations for the ‘Treasury of Human Inheritance’, initiated by Karl Pearson in 1909 and developed extensively over the next half century by his colleague Julia Bell [4].

By the time that Bateson published the definitive edition of his ‘Mendel’s Principles of Heredity’, also in 1909 [5], there was abundant observational evidence on the basic patterns of human inheritance, yet soon the momentum would shift to America with the beginnings of experimental research on Drosophila by Thomas Hunt Morgan and his colleagues. In 1914 European work came to an almost complete halt with the outbreak of war, while American research could continue largely unhindered.

**Fig. 1:**
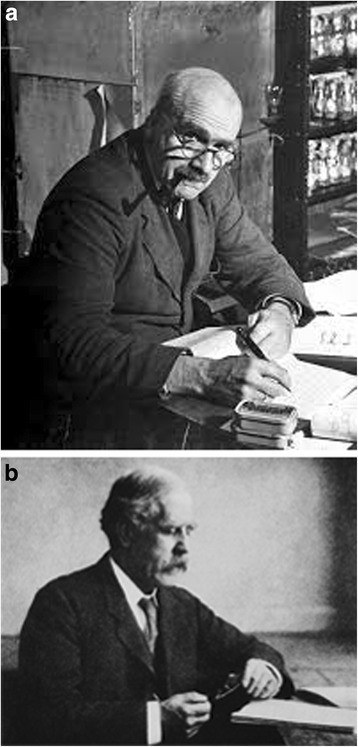
Early workers in human genetics and World War 1 (see text for details). **a**. JBS Haldane (1892–1964), pioneer of formal and quantitative human genetics (courtesy of Professor Peter Kalmus). **b**. Archibald Garrod (1856–1936), originator of the concept of ‘inborn errors of metabolism’. (Courtesy of Oxford University Press)

In personal terms the effects of World War 1 were devastating, though for genetics the situation was probably little different from other areas of science. JBS Haldane’s publication of the discovery of genetic linkage in mammals (mice) was delayed by his co-author Sprunt being killed in battle; Haldane himself (Fig. 1a) was severely wounded and, fearing that he also might not survive, had to write to his friend and colleague William Bateson:

‘*If I am killed could you kindly give my sister help if she wants it?’* [6].

Haldane’s remaining colleague in the study was his sister Naomi, better known now as the author Naomi Michison. In the event, Haldane survived and the paper was duly published, possibly the only such paper to be submitted from the battlefield (Haldane et al. [7]).

Archibald Garrod (Fig. 1b) lost all of his three sons in the war, two in combat, the third in the 1918 influenza pandemic; according to his biographer Alexander Bearn he never recovered from this blow (Bearn [8]), though he went on to produce what is perhaps his most insightful book, ‘The Inborn Factors in Disease’ (Garrod [9]) late in his life. There must have been many other such tragedies across the world. In France one of the few early proponents of mendelism, Lucien Cuénot, had all his research stocks of mice destroyed and was unable to continue work on genetics.

**Fig. 2:**
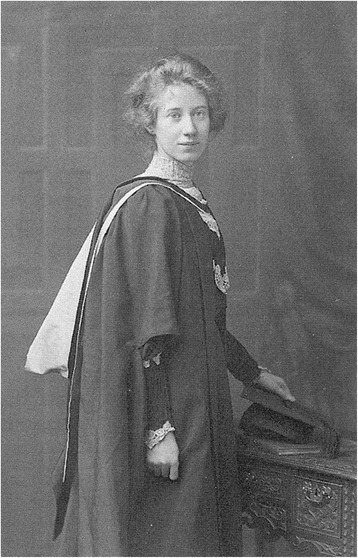
Julia Bell (1879–1979), principal author of the *Treasury of Human Inheritance*; originally trained in mathematics, but gained a medical qualification during World War 1. (Courtesy of Journal of Medical Biography and the late Professor Sarah Bundey)

There were some small positive notes to come out of the conflict. In Britain, Julia Bell (Fig. 2) took the opportunity of most men being away in the armed forces, to train and qualify in Medicine; she had until then been finding it difficult, as a mathematician working on the ‘Treasury of Human Inheritance’ with Karl Pearson, to gain the cooperation that she needed from the physicians (Harper [4]). And on the Macedonian front of the war, the husband and wife team of the Hirszfelds, working in the blood transfusion service, were the first to recognise ethnic differences in blood group frequency between the different population groups involved (Hirszfeld L and Hirszfeld H [10]).

**Fig. 3:**
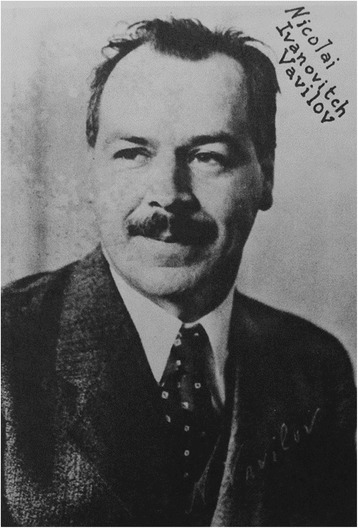
Nikolai Vavilov (1887–1943), plant and evolutionary geneticist; promoter of genetics overall in the new Soviet Union. (Courtesy of the John Innes Foundation)

The first decades following the end of the war provided a respite during which European science could recover and begin to advance again, while awareness of the importance of genetics spread rapidly across the world from the initial centres in Britain, America and Scandinavia. Human genetics was prominent in the work of more general geneticists such as JBS Haldane and Lancelot Hogben in Britain, while workers such as Norwegian physician Otto Lous Mohr also spent time studying classical genetics with Morgan and Sturtevant. In Russia the brilliant plant geneticist Nikolai Vavilov (Fig. 3), who had studied in Britain with Bateson (and had almost died on his return journey when his ship was sunk at the beginning of World war 1) had developed an extensive network of research institutes across what had now become the USSR. He had also made links with Hermann Muller in America and had sent students to train with him, including Solomon Levit who, as described below, was to become director of the remarkable Moscow Institute of Medical Genetics. By the early 1930s genetics, with human genetics prominent within it, had become a flourishing science internationally, with a closely knit research community.

The respite would not last long however, and during the 1930s two different, though not entirely separate shadows were beginning to fall across the field; in Germany Hitler’s coming to power in 1933 led at once to the dismissal and in many cases expulsion of Jewish scientists from the universities, and to the ‘eugenics law’ of the same year. In Russia, the opposition of Stalin and his agents, notably Trofim Lysenko, to orthodox genetics led to its near total destruction. In contrast to the somewhat random destruction seen in World War 1, these two disastrous and long lasting chapters were largely focused specifically on genetics and geneticists, especially those involved in human genetics. I shall consider the Russian situation first.

## The destruction of Russian genetics

During the twenty or so years since Vavilov had returned to Russia from Britain, science in general and genetics in particular had made spectacular advances in the new USSR, largely due to funding support on a massive scale, with the backing of Lenin himself. The main focus was on plant genetics, understandably so given the problems posed by recurrent famine, the hostile climate and the backward attitudes of the Russian peasantry. When William Bateson visited his former student in 1925 he was astounded by the sheer scale of Vavilov’s operations – 400 research stations across the entire Soviet Union, with around 20,000 workers in all (Vavilov [11]). Vavilov himself had led a series of research expeditions to areas such as Central Asia, North Africa and South America, which he considered to be likely centres for the origins of the key domestic food crops, notably grains, and he set up a far sighted programme of seed banks and of breeding programmes from these expeditions. But Vavilov’s aims extended far beyond plant genetics and included the promotion of human genetics, notably by medically trained workers such as Solomon Levit, who became head of the new Moscow Medical Genetics Institute. There were also other distinguished workers in the genetics field, most notably Nikolai Koltsov, whose work in the area of cell biology and especially the roles and structure of macromolecules such as nucleic acids [12], can be regarded as forerunners in the 1930s of the much later workers in molecular biology.

One area of human genetics where Russian scientists were considerably ahead of their western counterparts was human cytogenetics. Here they had managed to overcome some of the major technological barriers that were holding back progress, anticipating the West by 20 years or more in the use of hypotonic solutions to spread chromosomes, and the use of mitotic stimulants to allow the chromosomes of peripheral blood to be studied, rather than being dependent on dividing tissues such as bone marrow or testis which required more invasive procedures to obtain them. But the range of studies was wide, spanning population genetics, statistical studies and analysis of families with mendelian disorders, as well as of common conditions such as diabetes, as can be seen in the four published volumes of ‘annual reports’ from the Moscow Medical Genetics Institute, edited by its director, Solomon Levit, with its final volume in 1936. Work from the Institute was also published in mainstream Western journals, making the Russian workers part of the international network of geneticists, while Levit and some others were allowed to travel abroad to spend a year or more working in the laboratories of eminent geneticists such as Hermann Muller. A sympathetic account of Levit’s life and tragic death has been written by Dalia Epstein, (though not yet published outside a museum bulletin). Born in humble circumstances to a Lithuanian Jewish family, he was an ardent communist and one of the many betrayed by the revolution that he had supported.

All of this immensely productive activity in Soviet genetics was cut off abruptly in late 1936, when politically based criticism of genetics that had been increasing for several years erupted into a full blown crisis. Organised by Lysenko and his colleague the political theoretician Isaac Prezent, but with the full support of Stalin from an early stage, a ‘debate’ was held, which served to identify and hence incriminate the key supporters of mendelian genetics, who clearly won the scientific arguments, but the political battle had been lost in advance. Like Lysenko, Stalin was a strong proponent of the inheritance of acquired characteristics, not just in plants but for humans also, where ideas of malleable inheritance were more congenial to communist ideology than were the more fixed concepts of inheritance based on genes and chromosomes. Lysenko himself was a largely uneducated plant breeder, whose work was later shown to be largely erroneous, unrepeatable when performed under rigorous conditions (see below) and probably fraudulent, and whose agricultural policy of ‘vernalisation’ of wheat, based on supposed modification of its inherited character by freezing and thawing prior to sowing, resulted in disastrous crop failures.

Although the experimental work on plants did not directly involve human genetics, this was in fact in the front line for criticism, partly because some of the early workers in the field, including Koltsov, had been previously associated with eugenics, usually in rather vague and idealistic forms. But by the mid 1930s the abuses of eugenics that were beginning to occur in Nazi Germany were becoming known in Russia, and the whole of classical genetics became tarred with the same brush. The situation was aggravated by the fact that Vavilov, after years of lobbying his friends in the West, had succeeded in persuading both the congress organisers and the Soviet authorities to have the 1937 International Genetics Congress held in Moscow. By 1936 the thought of having numerous independent minded and mostly critical international scientists visiting Russia was too much for Stalin and his colleagues; first all presentations involving human genetics were banned, then the programme and speakers were to be chosen by the Soviet delegation, and finally the Congress was completely cancelled at short notice.

A further factor that made matters still worse was the involvement of Hermann Muller (see Carlson [13]) [14], already world famous from his discovery of genetic mutations due to irradiation in Drosophila. Muller, like a number of other Western geneticists, was an ardent communist and supporter of Soviet Russia; his earlier turbulent history included dismissal from the University of Texas for supporting student activists, which led him to leave America and accept a post with the outstanding Russian geneticist Nikolai Timoffeef-Resovsky, who was at the time (1932) working in Berlin. But the coming to power of the Nazis the following year made Berlin impossible for Muller, who then took up an open invitation from Vavilov to move to Russia. Here he made an immediate and lasting mark, founding groups involved in mutation research that flourished. But genetics was becoming increasingly beleaguered and in a fatal misjudgement Muller decided in 1936 to write directly to Stalin, enclosing his recently published book *Out of the Night* (Muller [14]) [15], in part of which he expressed his utopian ideas, written many years before, on the benefits of eugenics, which he naively considered might flourish under the Soviet system. Stalin, an avid reader, had the book translated and was appalled by it, with the result that the sequence of events leading to the destruction of Russian genetics was set in motion.

**Fig. 4:**
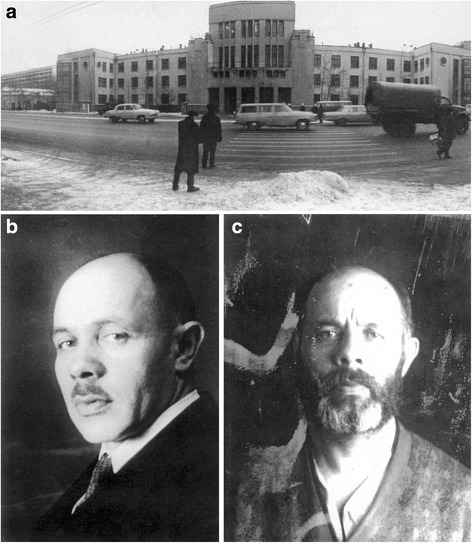
The destruction of Russian human genetics (photographs courtesy of Dalia Epstein, Lithuanian Historical Museum, Vilnius).**a**. The Moscow Medical Genetics Institute, 1934. The first and largest institute to be devoted to Medical Genetics; abolished 1937. **b**. Solomon Levit (1894–1938), first (and only) director of the Moscow Medical Genetics Institute. **c**. A prison photograph of Solomon Levit following his arrest in 1937 (precise date uncertain)

**Fig. 5:**
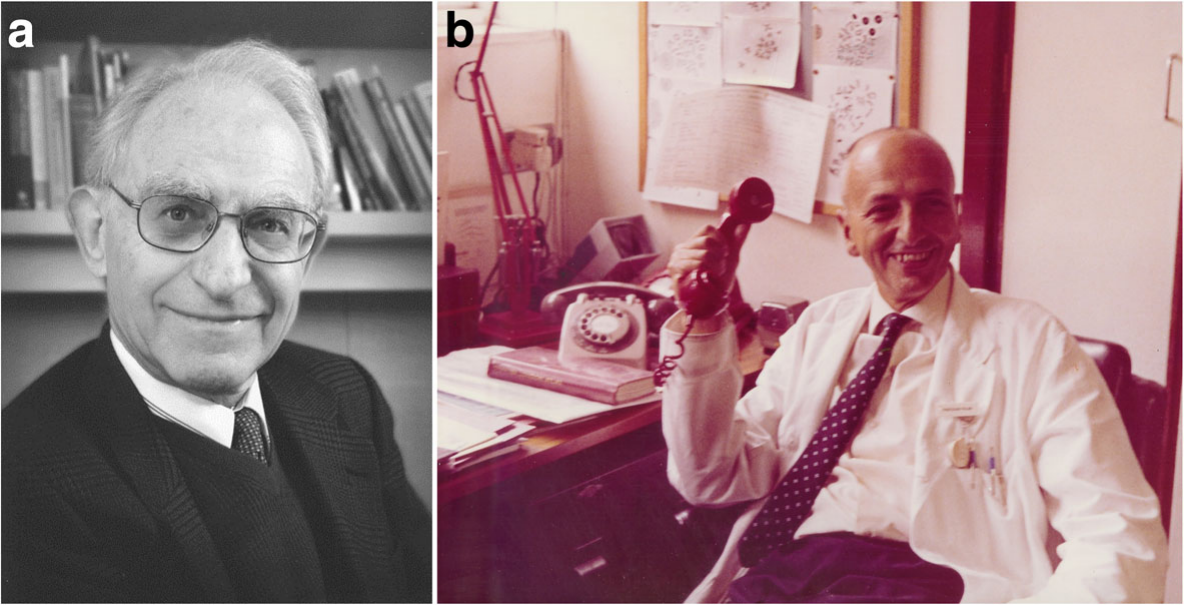
Refugees from fascism in Europe around the onset of World War 2. **a**. Arno Motulsky (born 1923); from Germany to (eventually) USA. (Courtesy of Dr. Arno Motulsky). **b**. Paul Polani (1914–2006); from Italy to UK. (Courtesy of Paediatric Research Unit, Guy’s Hospital, London)

Early in 1937 the Moscow Institute of Medical Genetics (Fig. 4) was abruptly closed, its director Levit (Fig. 5) was dismissed, imprisoned, and subsequently shot, as were a number of other geneticists; Muller, helped by Vavilov, escaped to join the Spanish civil war; Koltsov, after being dismissed from his posts, died suddenly (perhaps poisoned, perhaps a natural occurrence) and his wife committed suicide. Timoffeef-Resovsky remained precariously in Berlin until the end of the war, when he was arrested and placed in a concentration camp. Vavilov was dismissed but remained free until 1940, when he was arrested while on his final field expedition; he died from starvation in prison in 1943 despite repeated international calls for his release (Popovsky [15]) [16]. The 7th International Genetics Congress was finally held in August 1939 in Edinburgh, on the verge of the outbreak of World War 2; Vavilov had been elected President, despite no-one knowing whether he was still alive, and a symbolic empty chair was left for him on the podium. Russian genetics, including human genetics, had been essentially destroyed and would remain almost non-existent for the next 25 years.

## Human genetics and Nazi Germany

While the process of ascent followed by catastrophic fall was being played out so dramatically in Soviet Russia, human genetics was also involved in disturbing events in Germany; the key difference from the Russian tragedy is that geneticists were not the victims but were among the perpetrators and abettors of some of the worst crimes the world has ever seen. I do not attempt to discuss this chapter of events, or the topic of eugenics here in any detail, but it cannot be overlooked.

Around the time of World War 1 Germany was in the forefront of genetics research, as in most other fields of science, and in 1921 three of its leading geneticists, Erwin Baur, Eugen Fischer and Fritz Lenz, published what rapidly became the standard book on human genetics, *Menschliche Erblichkeitslehre*, translated into English in 1931 under the title *Human Heredity* [16]. The book ran to several editions and was apparently read by Adolf Hitler while in prison during the 1920s. At first sight the book appears to be a fairly standard textbook, giving a thorough and up to date grounding in the subject, but in some long chapters, mainly on anthropology, scientific description is mixed with prejudicial comments on racial characteristics and their superiority or inferiority, which increase and alter with successive editions.

Both Lenz and Fischer were Nazi party members, as were some other leading German geneticists, including Ernst Rudin and Otmar von Verschuer, so it is not surprising that when the ‘Law for the prevention of progeny with hereditary defects’ was enacted in July 1933, only six months after the Nazis came to power, its foundations had already been prepared, largely by the leading geneticists in the country.

Shortly before this, in April 1933, all Jewish University staff were dismissed from their posts immediately, leading to the flight of many of those able to leave the country (around 2600 in the first year alone) and to the catastrophic loss of talent from German science which took generations to repair. A remarkable book, *Hitler’s Gift*, (Medawar and Pyke [17]) gives details on the many talented scientists lost to Germany, and gained mainly by Britain and America, as a result. The book focuses on physicists, but there were a number of biochemists (eg: Max Perutz, Hans Krebs, Hermann Lehmann), as well as geneticists (Hans Grueneberg, Hans Kalmus, Charlotte Auerbach), who were found posts in London, Edinburgh and other British University centres, and who subsequently developed outstanding careers there. Those moving to America included Richard Goldschmidt and Curt Stern among others. Not all made the journey successfully, though. Arno Motulsky (Fig. 5a), who later in Seattle was to become one of the three key founders of North American medical genetics (along with Victor McKusick in Baltimore and F Clarke Fraser in Montreal), has told how his emigrant ship came into sight of the American coast but was refused entry and had to return to Europe, before he eventually reached America several years later [18].

The story of the finding of posts for these individuals in Britain by the Government-supported Academic Assistance Council is a heart-warming one, even though it can be regarded with hindsight as ‘enlightened self-interest’; people notably involved included JBS Haldane, then at University College, London, and William Beveridge, head of London School of Economics and subsequently the founder of Britain’s post-war ‘welfare state’.

Many of the Jewish refugees came with their young families, while a number of unaccompanied children came on the ‘Kindertransport’ trains from Berlin and Vienna immediately before the war began. It is this younger generation who make up a sizeable number of the post-war founders of human and medical genetics with whom I was able to make recorded interviews in my own series. Too young to be among those geneticists already established in their own countries, their lives were nonetheless profoundly affected by the war. I cannot give their stories in full here, but they can be read in the interview transcripts of the series, on the website of the Genetics and Medicine Historical Network (www.genmedhist.org/interviews) and they also receive mention in an article on the interview series as a whole [19]. The situation was strikingly comparable to that of today’s influx of refugees from across the world, and one wonders how history will judge our reception of these children by comparison with that of the earlier group.

Life was often far from easy for these refugees and on the outbreak of war a number of them were interned in a camp on the Isle of Man, initially alongside British Nazis. Some were then transported further to Canada, including those, mainly of Italian origin, on the ill-fated ship *Arandora Star,* which was sunk in the Atlantic with heavy loss of life. Paul Polani (Fig. 5b), one of the key founders of British medical genetics, who had come to Britain to undertake research and was then interned when Italy entered the war, narrowly missed being on the *Arandora Star* by being called to London to provide locum cover for a sick paediatrician, a position which turned out to last for the entire war and led to his subsequent career in medical genetics at nearby Guy’s Hospital. Ursula Mittwoch, who had come with her parents from Berlin just before the war aged 15, found herself interned as soon as she reached her 16th birthday. Later she made a distinguished career at University College, London.

The Isle of Man camp is documented in the book *Island of Barbed Wire* (Chappell [20]) but is perhaps described most memorably in molecular biologist Max Perutz’s essay ‘Enemy Alien’. This reflects his stoicism and humour in adversity and his recognition that most of what ill treatment there was came from bureaucracy and confusion as to who this heterogeneous assembly of internees actually were. As Perutz comments [21], on his way to the Isle of Man:

*Our camp commander was a white-moustached veteran of the last war; then a German had been a German, but now the subtle distinctions between friend and foe bewildered him. Watching a group of internees with skullcaps and curly side-whiskers arrive at his camp, he mused, ‘I had no idea there were so many Jews among the Nazis’. He pronounced it ‘Nasis’.*

Continental Europe, rapidly overrun by Hitler’s troops, was no refuge for Jewish or anti-fascist geneticists, and many must have perished. Among them was Eugene Wollman, microbial geneticist and father of Elie Wollman, molecular biologist colleague of Jacques Monod and François Jacob, both of whom fought with the French resistance, Monod himself helping to lead the liberation of Paris by its inhabitants. Among younger French workers that I have been able to interview, biochemical geneticist Jean-Claude Kaplan had to live under an assumed identity in occupied France, as also did Robert Debré, founder of post-war medical genetics in France.

## World war two

The 7th international Genetics Congress, cancelled by Moscow, as already mentioned, eventually met in Edinburgh in 1939, on the eve of the outbreak of war; it had to be concluded early, but it left a permanent memorial in the form of the *Geneticists’ Manifesto*, drafted primarily by Hermann Muller (now back from Spain and working in the Edinburgh department of Frank Crew), but signed by a number of other prominent geneticists (anon 1939) [22]. Coupling the science of genetics firmly to issues of social progress and freedom, the Manifesto at least ensured that the future of human genetics could be seen as a progressive and enlightened one, not tied for ever to the reactionary forces of eugenics and Nazi Germany. There was to be much strife, though, before the challenges of the Manifesto could be picked up at the end of the conflict six years later.

A reminder that the world was now at war came rapidly, with the sinking in mid Atlantic of the ship *Athena* carrying many of the returning American congress participants, as recounted by Arthur Steinberg, who was travelling in a separate ship that helped to rescue survivors (Jenkins [23]). Some of the Polish geneticists remained in Edinburgh permanently, their home country now invaded and partitioned as a result of the Nazi-Russian pact. For the following months the ‘phoney war’ gave an illusion of calm across Western Europe, at least on land, but this was abruptly shattered by the German ‘blitzkrieg’ on the Low Countries, Scandinavia and then France.

A geneticist’s viewpoint of these dramatic events can be seen from the perspective of Norway, where classical genetics and a considerable amount of human genetics research were flourishing in a modest way under the leadership of Otto Lous Mohr, a physician and geneticist trained in America under Thomas Hunt Morgan. The genetics community there had closed ranks firmly to oppose the eugenic and pro-Nazi views of some anthropologists, notably Alfred Mjoen, and had invited Lancelot Hogben from Britain, also outspoken in his opposition to eugenics, to give a lecture in Oslo. The minutes of the meeting have been preserved. Next morning, while Hogben (with his daughter) was being driven to the airport to return to London, they encountered columns of German troops, part of the invading force, but fortunately they were able to divert and travel on a remote road across the border with neutral Sweden, where he was looked after by his friend Gunnar Dahlberg, another committed anti-eugenicist. It took him almost three years to get home, though, after travelling East through Russia, on the trans-Siberian railway, to Japan and then across the Pacific to America [24]. Back in Norway, Mohr was dismissed and imprisoned under the puppet ‘Quisling’ regime, but later became Rector of Oslo University and survived to see his nephew, Jan Mohr, become one of the leaders of post-war European Human Genetics.

The wartime role of Jacques Monod and Francois Jacob in France has already been mentioned. Jacob was severely wounded in the Normandy landings, ending his hopes of becoming a surgeon but allowing him to become one of the leaders of molecular biology. His future colleague Jacques Monod had an even more dramatic role, becoming leader of the communist led underground resistance that led to the liberation of Paris in 1944. Almost unbelievably he managed to combine this with continued research at Institut Pasteur. Horace Judson [25], in his book ‘The Eighth Day of Creation’, gives a vivid account of these remarkable years, based on numerous interviews with those involved.

**Fig. 6:**
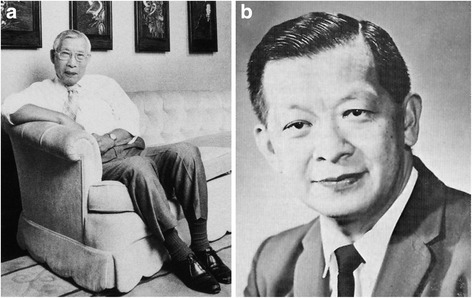
Human geneticists in the far East and World War 2 (see text). **a**. CC Li, 1912–2003 (China). (Courtesy of American Society of Human Genetics). **b**. J-H Tjio, 1919–2001 (Indonesia). (Courtesy of Professor Henry Harris)

On the other side of the world geneticists were no more immune to catastrophe than their European counterparts. China had already been invaded by Japan, largely destroying the promising beginnings that were developing largely as the results of links with America. Thus population geneticist CC Li (Fig. 6a), after studying agriculture and genetics in Nanking, came to Cornell University for a PhD, returning to China under exceptionally difficult wartime conditions, including a 38 day walk together with his pregnant wife, eventually reaching Kweilin, beyond the area of Japanese control. After the war he was appointed as professor at Peking University, where he wrote his book on human population genetics, but following the communist revolution in 1949, which brought with it Russian Lysenkoist doctrines, he was forced to leave. The rest of his life was spent based at University of Pittsburgh (Spiess [26]).

Another scientist of Chinese origin who suffered lasting scars from the war was Joe Hin Tjio (Fig. 6b), brought up in Indonesia and interned and tortured successively under the Japanese during the war and then as a communist supporter under the new government. Eventually he made his way to the Netherlands, being awarded a scholarship to study genetics across Europe; this allowed him to establish a university base in Zaragosa, Spain as well as to make links with Albert Levan in Lund, Sweden, where he spent summers and vacations. This collaboration led to the realisation that the human chromosome number was 46, not 48 as had been erroneously believed for the previous 30 years (Tjio and Levan [27]). Tjio’s later years were spent in America, mainly at the National Institutes of Health.

In Japan human genetics had also made a promising start in the 1930s, notable contributions including the recognition of mitochondrial inheritance as the basis for Leber’s optic atrophy (Imai and Moriwaki [28]). The war halted this progress, not only in Japan itself, but also in America, where after the Pearl Harbor bombing, Japanese-American citizens were interned in the same way as their European counterparts in Britain, including cytogeneticist Masuo Kodani (1913–1983). The profound consequences on Kodani’s life and career are described by Smocovitis [29] and the entire internment process appears to have been more inhuman than that in Britain, though several internees, including Kodani, managed to continue valuable cytogenetics research in collaboration with outside scientists. But Japan’s principal role in human genetics has resulted from the terrible blow suffered by the populations of Hiroshima and Nagasaki when the atomic bombs were dropped that ended World War 2. By this time it was already recognised, thanks to the work of Muller and others, that irradiation could cause genetic mutations, but little was known about how this might translate into genetic disorders and birth defects in a real population.

With the war’s abrupt end both politicians and geneticists recognised that the dangers from radiation threatened not only Japan’s bombed cities but the entire world; the practical result of this concern was the American led ‘Japanese Atomic Bomb Study’, which aimed to detect and as far as possible estimate the genetic effects of the explosions. Very wisely, and perhaps unexpectedly so soon after a war, the American organisers decided to bring Japanese scientists into partnership for the study, not only facilitating the project but stimulating a strong scientific tradition for Japan in radiation genetics and human cytogenetics that continues to the present.

**Fig. 7:**
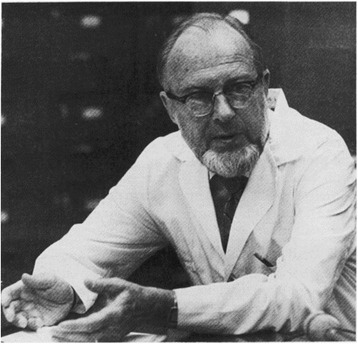
JH Neel (1915–2000), leader of the US atomic bomb genetics studies. (Courtesy of American Philosophical Society)

The project director was James Neel (Fig. 7), a physician and geneticist who saw that much more needed to be known about human genetics in general if the Atomic Bomb Study were to be interpreted meaningfully. This was the start of the close and long lasting links between human genetics and radiation biology that resulted in rapid growth of the field across the world over the next two decades, as described further below. There were wider benefits, too, since some of the American investigators, based in Japan for a number of years, became closely identified with Japanese culture and social life, helping to restore, at least in a small way, the links broken by the war. The book *Song among the Ruins,* by geneticist William Schull, (Schull [30]) provides perhaps the best example of this.

## Aftermath: the post-war rebirth and development of human genetics

### Radiation and human genetics

The years after the end of World War 2 were not easy ones, whether for scientists or for people as a whole. In Europe especially, reconstruction, both physical and social, took priority over other matters, though the foundations that were laid for universal healthcare in countries such as France, Britain and the Netherlands greatly facilitated the development of medical genetics services two decades later. Research funding was rapidly restored, with the dangers from irradiation making human genetics a priority area. This requires further discussion here, and is an important general historical topic too.

It rapidly became clear, as already mentioned, that the problem of atomic radiation was a world-wide one. Atmospheric fallout from nuclear testing, the proliferation of nuclear weapons and the growing ‘cold war’ all gave intense political as well as public concern, so that genetics, and especially human genetics, became a strong focus for research institutes worldwide. This also affected the university systems, with chairs in human genetics established and funding of projects only tenuously related to radiation. The First International Human Genetics Congress, held in Copenhagen in 1956, likewise laid a heavy emphasis on the genetic dangers from radiation.

The UK Medical Research Council’s two main units concerned with radiation, in Edinburgh and Harwell, provide a good example of how radiation related research benefited human genetics as a whole, by helping to develop new techniques such as the analysis of human chromosomes, and also by their foresight in supporting work which might have seemed beyond their remit, such as studies of Down’s syndrome and of sex chromosome abnormalities. I have been able to interview a number of these workers (see www.genmedhist.org/interviews) including Patricia Jacobs, Mary Lyon and Anthony Searle, and the interview transcripts provide a striking example of the freedom that they had in the range of their research topics.

The situation in Russia provides the most extreme example in this field of radiation genetics. Despite Lysenko’s suppression of genetics in the research institutes, and after 1948 in the universities also, he had no authority over one area, that of atomic energy, where in the desperate haste to catch up with the West its directors had ‘carte blanche’ to employ who they wished and to undertake all types of research. Safety considerations were minimal or absent, and the situation became critical following a massive explosion of buried nuclear waste at the secret Urals atomic research station, which spread radiation over a wide area of Russia – but not outside its borders, thus avoiding international detection (Medvedev [31]).

**Fig. 8:**
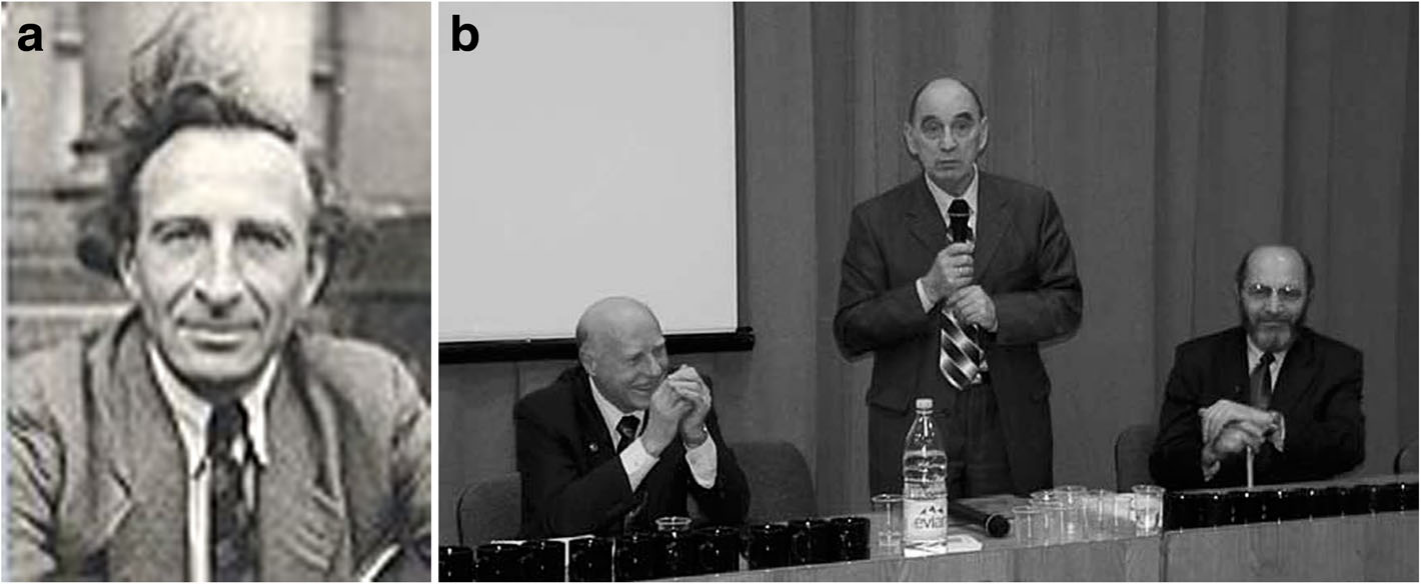
Survivors and re-creators of Russian human genetics. **a** Nikolai Timoffeef-Resovsky (1900–1981), one of the very few survivors of the destruction of Russian genetics by Stalin and Lysenko. (Courtesy of Professor V Ivanov). **b** Nikolai Bochkov, Yevgeny Ginter and Vladimir Ivanov, the first three directors of the renewed Moscow Medical Genetics Institute, all former students of Timoffeef-Resovsky. (Courtesy of European Society of Human Genetics)

There was clearly a need for information on likely short- and long-term effects, especially genetic damage, but there were virtually no scientists left with expertise in this area since Lysenko and Stalin’s purges of geneticists, which had continued after the war. The only suitable survivor was Nikolai Timoffeef-Resovsky (Fig. 8a), mentioned earlier, who had been brought back from Berlin and imprisoned. Close to death, he was released, restored to tolerable health and placed in charge of a new radiation research unit, whose staff, bizarrely, were all political prisoners, including himself.

Subsequently Timoffeef-Resovsky, still distrusted politically, went to Obninsk, not far from Moscow, where he led a unit which provided the tenuous beginnings for a revival of human genetics at the end of the 1960s, after Lysenko had finally been discredited. The renewed Moscow Medical Genetics Institute has been directed successively by three of Timoffeef-Resovsky’s workers (Fig. 8b), a striking example of a ‘founder effect’ resulting from the elimination of most, but not quite all, of Russia’s geneticists.

Despite this focus on radiation genetics, Russia still seemed ill prepared for any practical applications when the Chernobyl disaster occurred in 1986. This was made clear to me in a recorded interview in 2005 with Gordon Laziuk, geneticist and paediatric pathologist in Minsk, who had established a congenital malformations register covering the affected region. (See www.genmedhist.org/interviews). Going at once to Chernobyl he found nothing but confusion and obstruction, as well as panic among the local population. The disaster had not been admitted until the radiation had spread beyond Russian borders; my translator for the interview commented to me that at his Moscow molecular genetics institute the first indication that there was any problem was when police came and removed all Geiger counters from the building! (Nikolai Yankowsky, personal communication 2005).

**Fig. 9:**
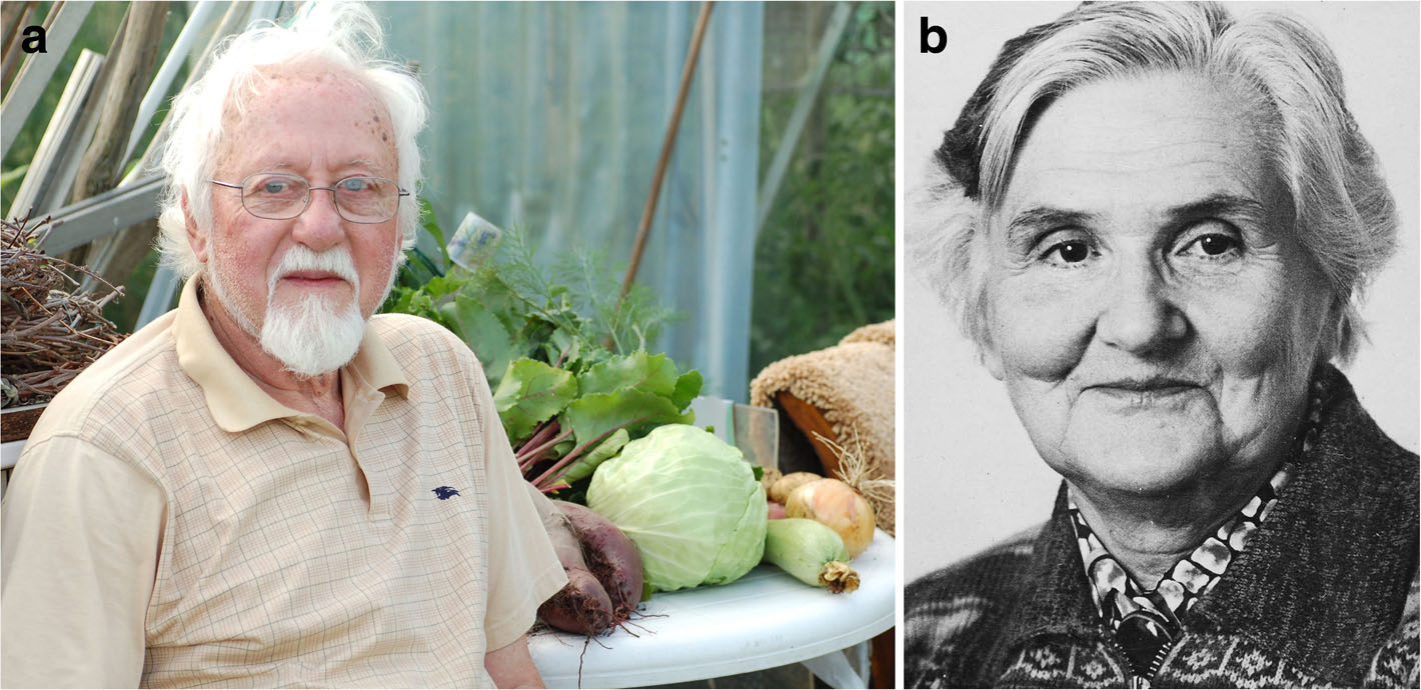
Geneticists exiled from the Soviet Union for writing the truth about the destruction of Russian genetics included: **a**. Zhores Medvedev (Born 1925). (Courtesy of Professor Medvedev). **b**. Raissa Berg (1913–2006). (Courtesy of Darhansoff Verrill Feldman literary agency).

The suppression of Russian genetics extended beyond workers in the field to include those who attempted to record and chronicle this bizarre and tragic chapter. Foremost among these has been Zhores Medvedev (Fig. 9a), yet another colleague of Timoffef-Resovsky in Obninsk in the late 1960s and well established in the field of radiation genetics and cell aging. He had witnessed the earlier destruction of genetics as a young worker and decided to put as much as possible on the record, now that Stalin was dead and Lysenko disgraced. His book, *The Rise and Fall of TD Lysenko* [1], gives a largely first-hand account of events; it was circulated through the ‘Samizdat’ underground network, publication in Russia having been banned, and after its English translation appeared Medvedev was first imprisoned in a psychiatric hospital and then expelled while on a research visit to London, where he spent the rest of his career, and where I was able to interview him in 2006. Others attempting to chronicle the past events followed a similar fate, including Marc Popovsky, Valery Soyfer, and most notably and recently Raissa Berg (Fig. 9b), a former student of Hermann Muller in Russia; any attempt at criticism of the past, particularly in relation to human genetics, was forbidden up to around 1990, when Mikhail Gorbachev as President allowed the topic to be opened up, leading to the 2005 book of Babkov, *The Dawn of Human Genetics*, translated into English in 2013 [32]. Certainly the tensions were still palpable at the 1978 International Genetics Congress in Moscow, the eventual successor to the ill-fated and cancelled 1937 congress.

### Eastern Europe and Lysenkoist genetics

When the ‘iron curtain’ descended in 1945 to cut off the countries of Eastern Europe from the rest of the continent, Russian dominance also meant that Lysenkoist doctrines were imposed on geneticists, with traumatic results. Geneticists in Eastern Europe also became largely isolated from colleagues and research networks in the West, and many were dismissed or forbidden to teach. But some of these countries, notably Czechoslovakia, had a well-established, lengthy and proud tradition of classical genetics, beginning with Mendel himself, something that could not easily be eradicated. Also the isolation was never complete, especially in Germany, where Berlin initially provided a porous border between East and West and where even the communist authorities were far from convinced by Lysenko’s ideas. In East Germany a key figure was Hans Stubbe, a plant geneticist based in Halle, whose opposition was based on subversion rather than on direct resistance. Taking the line that Lysenko was bound to be correct but that more facts confirming his theories and disproving mendelian genetics were needed to convince the rest of the world, he and his colleagues repeated the experimental work under carefully controlled conditions. When the results turned out consistently to be the opposite of what was officially expected, this seriously undermined the standing and validity of Lysenkoist genetics. Stubbe’s key role is described by Rudolf Hagemann [13], who worked with him in Halle. In fact East Germany had quietly reverted to orthodox genetics some years before this occurred in Russia. In later years West German human geneticists made determined efforts to increase links, as Friedrich Vogel describes in his 2003 recorded interview with the author (www.genmedhist.org/interviews), in relation to the 1986 Berlin International Human Genetics Congress. A general account of the development of medical genetics and genetic counselling in the former East Germany has recently been given by Doetz [33].

In Czechoslovakia the process was more difficult, since Mendel, reviled as the origin of all matters relating to ‘capitalist genetics’, was still present in spirit in Brno; two interviews in my series, with medical geneticists Milan Macek (Prague) and Renata Laxova (Brno) illustrate some of the problems and tensions. Fortunately Lysenkoism had been abandoned shortly before the 1965 centenary of Mendel’s work, and the meeting to celebrate this in Brno [34] attracted many international geneticists who helped to restore the standing of Czechoslovak genetics.

The damaging effects of Russian influence extended beyond Europe and were marked in China, as already mentioned; here, no sooner had this problem been overcome than Chinese geneticists were subjected to the persecutions of the ‘cultural revolution’, which affected many of the older workers still active today. They were also much involved in the arguments surrounding the ‘maternal and child health law’, [35] originally termed the ‘eugenics law’, parts of which, notably the list in the initial version of proscribed disorders for reproduction, were very similar to the 1933 Nazi eugenics law. This law was eventually passed in 1986 in the face of opposition from many Chinese human geneticists and from international protests, but it was later largely abandoned, or at least not implemented. Understandably this remains a sensitive subject in China and the full story of the background and prolonged internal discussions has yet to be written, though I have tried to put together some fragments gleaned as a result of my own peripheral involvement [36]. Now that China has become a leading player in genomics and restrictions on writing about uncomfortable topics have been loosened to some extent, it is important that this chapter of events is fully documented and recognised, since most young workers in the field are unaware of it.

A word must be said here about how western geneticists reacted to Lysenkoism. Most were horrified and there were impassioned defences of Vavilov and other persecuted Russian geneticists. But those who were committed communists faced a dilemma in who to support. Muller, with first hand experience of the situation, immediately supported orthodox genetics, though his public stance was more guarded to protect his Russian former colleagues. Jacques Monod made an outspoken denunciation of Lysenko and left the French communist party. Others, not to their credit, were more ambivalent, notably JBS Haldane, while molecular scientist and crystallographer Desmond Bernal totally denied that there was any problem in accepting Lysenko’s views, despite the work of his close colleagues on the structure of DNA. Historian Diane Paul [37] has written a valuable article on the dilemmas faced by these Western workers in relation to Lysenkoism. This episode shows how for some scientists communism had assumed the status of a religion, where facts had little influence on entrenched belief.

### The spread of human genetics across Europe and beyond

Restoration of progress in West European human genetics after the war was uneven but surprisingly rapid, with generous funding undoubtedly a factor in many countries. Centres such as London’s Galton Laboratory, with Lionel Penrose as head, acted as foci for training of people in the field and for more general influence, while others spent time at American centres, especially as medical genetics began to evolve from the basic science of human genetics. The patterns of development varied considerably between countries; thus in France, it was paediatricians who led the new field, its founders there, such as Robert Debré and Maurice Lamy, seeing that genetic disorders of childhood and congenital malformations were replacing infectious and nutritional diseases as the main causes of childhood mortality. In Britain a wider range of clinicians, from adult as well as paediatric backgrounds, became medical geneticists, a factor that helped to extend the scope of the field as specialists such as oncologists and neurologists became increasingly aware of genetic aspects and sought the help of medical geneticists. During the 1970s and 1980s most of the clinicians involved in the field became full time medical geneticists; defined training programmes were developed, while medical genetics itself became recognised as a distinct medical specialty in an increasing number of countries.

In 1967 the European Society of Human Genetics was founded and held its first meeting in Copenhagen; from the outset it was concerned to strengthen links among geneticists across the continent, especially with the recovering but still isolated workers in East Europe [38]. Its founders, notably Jan Mohr in Copenhagen, were keenly aware of the difficulties of their East European and Russian colleagues, and efforts to include them in meetings and later in collaborative studies proved an important factor in helping them rejoin the mainstream of human genetics.

Understandably the country for which human genetics has been and remains an especially sensitive and at times traumatic subject is Germany, in particular the former West Germany. This was aggravated by the post-war reappointment of a number of geneticists deeply complicit in the Nazi atrocities to important university positions, Otmar von Verschuer notable among them. These workers largely carried on with their previous research as if nothing had happened, as shown in the interviews in the book ‘Murderous Science’, by Benno Mueller-Hill [39], himself a bacterial geneticist, which led to considerable controversy and to the breaking away of most younger geneticists to form a new professional society. Petermann [40] has described the post-war development of human genetics across the Federal Republic of Germany.

## Conclusion: Human and medical genetics today and in the future - still dangerous times?

The beginning of the twentieth century saw the birth of modern genetics, and throughout the century dangers of one kind or another were closely associated with the new field, as we have seen. Will we see a continuation or repeat of these dangers across the world in the twenty-first century? While any attempt at prediction may be fruitless, we can at least try to identify those aspects of human genetics which might especially be associated with danger, and to recognise any potential warning signs. I shall try to do this here, though from the sidelines, as one no longer actively involved in clinical or research activity in the field, except from a historical perspective.

First, one must recognise that the field of human genetics itself has changed radically since its emergence from more general genetics some 70 years ago. There is a much larger body of workers involved, spread more widely internationally, and with a much larger proportion involved with medical aspects of genetics.

As Victor McKusick remarked in his 1975 address to the American Society of Human Genetics [41]:

*In the 1950s we heard some of our colleagues in biology bemoan the difficulties of stimulating interest in genetics on behalf of their medical school colleagues, and their complaints were well grounded in many instances. In the 1960s we heard some of them bemoan the taking over of the field by the medical school faculty. In the 1970s let us hope we are achieving a state of mutual respect and intimate collaboration between the two cultures.*

This body of workers includes not only medical geneticists themselves but specific non-medical genetic counsellors, particularly in North America and UK; a high proportion of both categories are women, especially well placed to understand the practical and emotional issues related to genetic testing and the impact of inherited disease. Laboratory scientists include many diagnostic workers and researchers into genetic disorders, as well as those involved with the more basic aspects of human genetics.

All this seems (and is) very different to the small groups of basic biologists who in the first half of the twentieth century were the self-appointed spokesmen on topics such as eugenics, and who discoursed on the supposed genetic problems associated with the poor, and with immigrant and minority ethnic groups.

A second factor that is likely to be a powerful defence against the widespread misuse of genetics is the incorporation of strong ethical principles into the practice of both human genetics research and medical genetics practice. In part this can be seen as the application of the general principles that evolved as a reaction to the atrocities of world war 2, but it is also the result of the development of a strong ethical dimension within medical genetics itself, instilled initially by a small number of key founders such as Lionel Penrose in Britain and Arno Motulsky in America, and incorporated into the fabric of human and medical genetics generally as the field has developed.

Finally, recent genomic research has increasingly shown that human populations are not only remarkably variable within themselves, but that supposedly different groups are for the most part remarkably similar genetically. Even with the worrisome rise of antagonism between different religious and social groups, there is no evidence that this is in any way mirrored by their genetic composition, while up to the present there have been few attempts to invoke or invent such genetic differences as possible factors in social conflicts. In contrast to the political involvement of the early twentieth century eugenicists, there are few or no geneticists now involved in making such claims.

On the negative side, the greatly increased power of modern genetic techniques gives a potential for abuse that greatly exceeds what was available to the dictators and totalitarian societies of 70 years ago. Computerised DNA databases, predictive testing for late onset disorders, genetic screening and genome sequencing are all areas capable of abuse to a much greater extent than was the minimal and largely fallacious scientific underpinning of eugenics in the 1930s, while many politicians across the world now show at least as much disregard for the truth as at that time. New reproductive technologies continue to produce ethical dilemmas that have no easy answers, especially when linked to commercial pressures.

Any optimistic conclusion to this article must therefore be tentative and cautious, given the numerous very real ethical issues and potential for abuse that do exist in the field of present day human genetics and genomics, both in research and in its applications. There are many political and other groups eager to misrepresent and distort sensitive advances, and it will require both vigilance from people in general and an active awareness and involvement of geneticists in the social consequences of new developments to ensure that this does not happen. Already there are early signs of such abuse, such as proposals for compulsory DNA testing of the entire population (Kuwait) and access to and use of genetic testing data by employers (USA).

Apart from the united and immediate condemnation of these and other abuses by the genetics community that is essential, a continued strong ethical stance, transparency and good communication with the public, avoidance of false or exaggerated claims, the maintenance of close international links, and self-criticism within the human genetics community itself, are just some of the factors which we, as human geneticists, will need to ensure remain at the centre of our field, and are transmitted to the younger generations of workers.

A vital part of this process is awareness of what has happened in the past, including both where human geneticists have failed in their responsibilities and where they have themselves suffered, sometimes at the cost of their lives, from their attempts to uphold the truth and an ethical approach to their research and practice. I hope that this article will contribute to such awareness world-wide, both among geneticists and among all those whose lives are affected in any way by the increasing applications of genetics to everybody.
